# Optical properties of exfoliated MoS_2 _coaxial nanotubes - analogues of graphene

**DOI:** 10.1186/1556-276X-6-593

**Published:** 2011-11-15

**Authors:** Bojana Visic, Robert Dominko, Marta Klanjsek Gunde, Nina Hauptman, Sreco D Skapin, Maja Remskar

**Affiliations:** 1 Jozef Stefan Institute, Jamova cesta 39, Ljubljana, 1000, Slovenia; 2 National Institute of Chemistry, Hajdrihova 19, 1000 Ljubljana, 1000, Slovenia; 3 Centre of Excellence Namaste, Jamova 39, Ljubljana, 1000, Slovenia

**Keywords:** molybdenum disulfide, nanotubes, exfoliation

## Abstract

We report on the first exfoliation of MoS_2 _coaxial nanotubes. The single-layer flakes, as the result of exfoliation, represent the transition metal dichalcogenides' analogue of graphene. They show a very low degree of restacking in comparison with exfoliation of MoS_2 _plate-like crystals. MoS_2 _monolayers were investigated by means of electron and atomic force microscopies, showing their structure, and ultraviolet-visible spectrometry, revealing quantum confinement as the consequence of the nanoscale size in the *z*-direction.

## Background

In recent years, significant progress has been made in exfoliating graphene directly from graphite, which is supposed to produce samples with fewer defects [1]. Exfoliation of the metallic-layered compounds TaS_2 _[2,3] and NbS_2 _[3] is known for more than 30 years. Also, preparation of a single molecular layer of MoS_2 _out of the crystalline 2H-MoS_2 _by intercalation of lithium has been reported in 1986 [4], which was the first exfoliation of a layered semiconductor, and it was followed by the exfoliation of WS_2 _[5]. Exfoliation via other solvents [6] and cleaving processes [7] has been reported recently. Until now, there have been no reports on the attempt to exfoliate transition-metal disulphide nanotubes.

Bulk 2H-MoS_2 _is made of S-Mo-S sandwich layers, where every molybdenum sheet is between two sheets of sulfur. It was found that crystalline MoS_2 _has three polytypes: 1T, 2H, and 3R, where the integer indicates the number of layers per unit cell and T, H, and R indicate the trigonal, hexagonal, and rhombohedral primitive unit cells, respectively. Whereas the interactions within the sandwich correspond to the chemical bonds, the neighboring layers are weakly connected with Van der Waals bonds, and foreign materials can be inserted into the Van der Waals gap, and under appropriate conditions, the layers can be further separated to form single molecular layers.

Single molecular layers of MoS_2 _in a water suspension have been prepared by intercalation of lithium into crystal 2H-MoS_2 _followed by exfoliation in water. As this aqueous suspension is aging, restacked MoS_2 _with two monolayers of water is formed (the water-bilayer phase), with water monolayers between parallel, but rotationally disordered MoS_2 _layers. For this structure, a 2*a*_0 _× *a*_0 _pattern was confirmed [8], where *a*_0 _is the lattice constant of bulk 2H-MoS_2_. Single layer shows a change in lattice symmetry from 2H to 1T, and it is suggested that the change in coordination is electronically driven by Li electron donation to the MoS_2 _host [9]; this configuration is preferred because the electrons donated to the valence band in 1T configuration occupy a much lower level than the electrons donated to the conduction band of the 2H structure. It was shown that this structural transition is followed by a change in the optical absorption spectrum, where two strong absorption peaks for 2H-MoS_2 _are absent [10]. The structural transformation is also present in the formation of single molecular layers of WS_2_. Lattice constants in the basal (001) plane were found: for 2H-MoS_2 _crystal with a trigonal prism configuration, it is 3.162 Å; for Li-MoS_2 _crystal with an octahedral configuration, 3.6 Å; and for MoS_2 _single layer with an octahedral configuration, 3.27 Å [11].

Exfoliation of MoS_2 _can lead to the synthesis of many new materials, obtained by restacking the single layers with, for example, organic molecules [12-14]. It was discovered that MoS_2 _photoluminescence increases with decreasing layer thickness, the strongest is on single layer [15], which holds promise for new nanophotonic applications, and it was also realized as a field-effect transistor [16], which can be applied in new areas of optoelectronics.

MoS_2 _is also known as a solid lubricant which has been used in the industry for the last 60 years. At low-humidity conditions, it is possible to obtain a low friction coefficient of 0.05 [17]. Ultra-low friction of 0.003 was reported between MoS_2 _flakes and MoS_2 _surfaces [18]. The problem of edge oxidation and preservation of the flakes in parallel orientation with the surface with a low degree of restacking can be minimized with the reduction of thickness. It is desired to obtain the thinnest flakes possible, which can be achieved by exfoliating MoS_2 _coaxial nanotubes.

## Methods

The MoS_2 _coaxial nanotubes (Nanotul Ltd., Ljubljana, Slovenia) are synthesized by sulfurization transformation of M_6_S_2_I_8 _nanowires under gas flow of H_2_/H_2_S mixture in an argon atmosphere [19]. They were dried in a dry box (glove box, < 1 ppm of H_2_O, M.Braun Garching, Germany) for at least 6 h in vacuum at 120°C, then suspended in a solution of 2.5 M butyllithium in hexanes (0.693 g/mL, Sigma-Aldrich, St. Louis, MO, USA), where it was left for 3 days. Exfoliation occurs by immersing the lithium-intercalated compounds in water after taking them out of the dry box, which provides a water-bilayer phase of MoS_2_. To obtain single layers of MoS_2 _in water suspension, the material was washed repeatedly with distilled water and centrifuged. The reaction that occurs between the water and intercalated lithium results in hydrogen gas release and lithium hydroxide formation. The washing process reduces lithium concentration (from a pH of 12 to 7). Consequently, the water-bilayer phase, which is stable in a higher pH [5], splits into single MoS_2 _layers.

The exfoliated material was characterized by scanning electron microscopy [SEM], transmission electron microscopy [TEM], atomic probe techniques (atomic force microscopy [AFM] and STM), and X-ray diffraction [XRD]. The XRD spectra were recorded with an AXS D4 Endeavor diffractometer (Bruker Corporation, Karlsruhe, Germany), with Cu Kα_1 _radiation and a SOL-X energy-dispersive detector with the angular range of 2*θ *from 5° to 75° with a step size of 0.04° and a collection time of 3 to 4 s.

The process of exfoliation was elucidated by ultraviolet-visible [UV-Vis] spectroscopy. The spectra were recorded in a 10-mm-path length quartz cell on an Agilent 8453 UV-Vis Spectrophotometer (Agilent Technologies, Inc., Santa Clara, CA, USA) at 23°C ± 1°C in a wavelength range of 180 to 1,000 nm, with a 1-nm resolution. For comparison, the PerkinElmer Lambda 950 photospectrometer (Waltham, MA, USA) was used under the same conditions. All UV-Vis measurements were performed on the material in a water solution.

## Results and discussion

**Figure 1:**
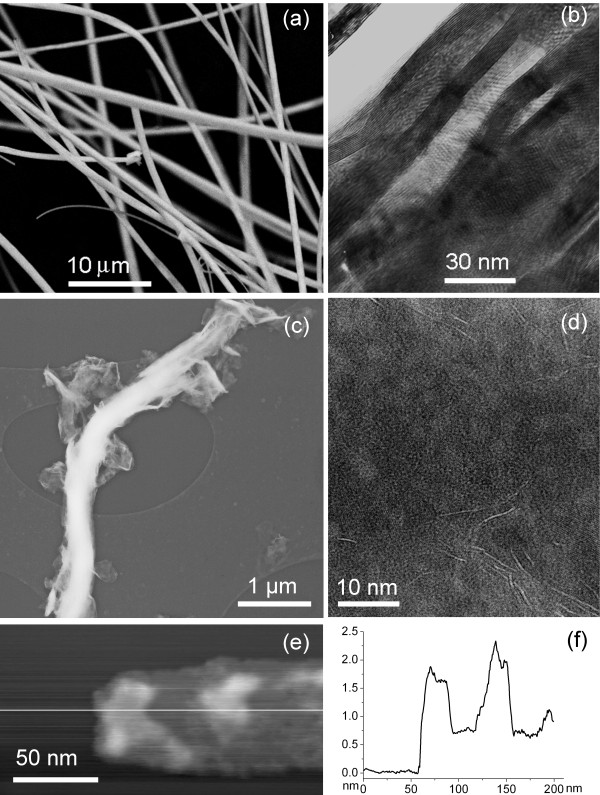
**Microscopy**. MoS_2 _nanotubes before exfoliation: (**a**) SEM image of short parts of millimeter-long nanotubes with diameters up to 500 nm; (**b**) TEM image of the pristine nanotube revealing spontaneous partial splitting of the nanotube's wall into several blocks, up to 10 nm in thickness; (**c**) TEM micrograph of the MoS_2 _nanotube during the first stage of the exfoliation process; (**d**) TEM micrograph of the MoS_2 _single layers as a final stage of nanotube exfoliation; (**e**) AFM image (contact mode) of the surface corrugation on a thin MoS_2 _nanoflake with a (**f**) corresponding line profile.

The main reason why the nanotubes (Figure 1a) are used for exfoliation is that they already have gaps between their coaxial cylinders, as shown in Figure 1b. The nanotubes keep an outside shape of the Mo_6_S_2_I_8 _nanowire precursor, but the difference in mass density between the wires and MoS_2 _compounds leads to a creation of an empty space inside the MoS_2 _nanotubes that separates the adjacent cylinders and creates gaps between them [19], so it is easier to intercalate them; and the nanotube walls have a curvature, which prevents restacking.

The TEM micrographs of exfoliated MoS_2 _nanotubes are shown in Figures 1c, d, while the AFM image and the corresponding profile are shown in Figures 1e, f, respectively. The final product of the exfoliation process contains mainly of single-layer MoS_2 _flakes.

The UV-Vis absorption spectra were measured on exfoliated MoS_2 _nanotubes, and the comparison was made with the MoS_2 _coaxial nanotubes (used for exfoliation) and 2H-MoS_2 _plate-like powder (< 2 nm, 99% purity, Sigma-Aldrich, St. Louis, MO, USA). The samples were prepared in a form of dispersion in water, with concentrations showing comparable intensities of optical absorption.

**Figure 2:**
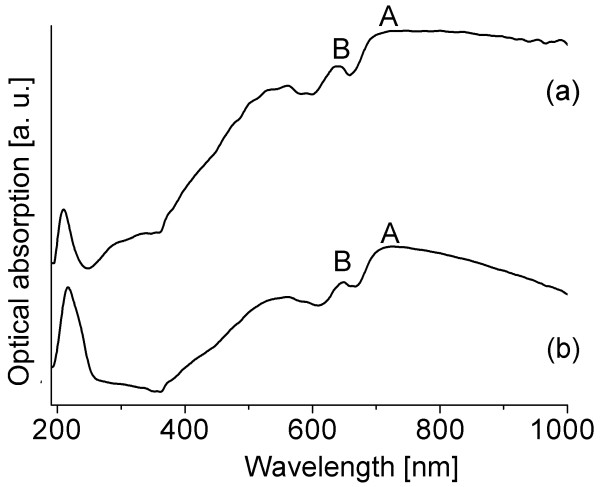
**UV-Vis spectra of the powder and nanotubes**. UV-Vis absorption spectra of the (**a**) MoS_2 _powder and (**b**) MoS_2 _nanotubes.

Both powder and nanotube spectra (Figure 2) show the features that can be assigned to the A and B excitons, characteristic for the 2H-polytype and correspond to the smallest direct transition at the K point of the Brillouin zone (K_4 _→ K_5 _and K_1 _→ K_5 _transitions, respectively) [20]. The existence of the two excitons is due to the interlayer interaction and spin-orbit splitting, with the splitting value of approximately 60 nm (0.17 eV).

For the MoS_2 _coaxial nanotubes, peak positions for A and B excitons are at 702 nm (1.77 eV) and 644 nm (1.92 eV), respectively, with the red shift of the absorption peaks compared to the powder, where their values are 692 nm (1.79 eV) and 634 nm (1.96 eV). The red shift is due to the quantum confinement, as explained by Frey et al. [21]. The excitons are separated by 60 nm for both materials, which is in good agreement with the literature [21]. Another broader peak, observed at 540 nm (2.30 eV), can be assigned to a direct transition between the states deep in the valence band to the conduction band at the M point of the Brillouin zone [20]. The strong peak at 210 nm (5.9 eV), being at the edge of the spectrometer's range, is usually disregarded from the analysis, for the wavelengths were so small, we get increased scattering, and one of the consequences is a false peak. Since the energy associated to this peak is too large to be indubitably assigned to a particular electronic transition, the nature of the peak is still inconclusive.

**Figure 3:**
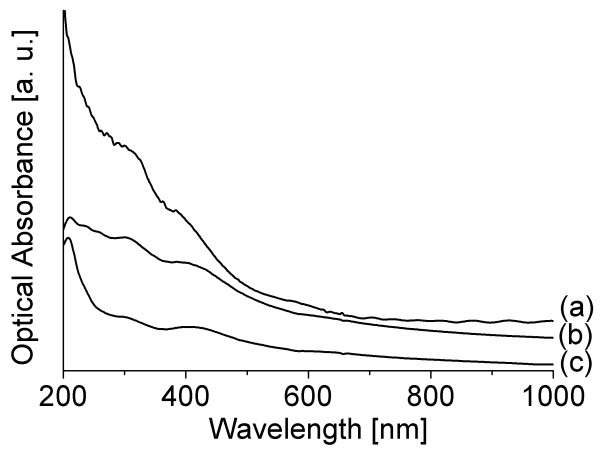
**Aging**. Absorption spectra of MoS_2 _during the exfoliation process: (**a**) the initial sample containing Li-MoS_2_; (**b**) the completely exfoliated MoS_2 _nanotubes; (**c**) the completely exfoliated MoS_2 _nanotubes after 29 days.

The change from Li-MoS_2 _to exfoliated MoS_2 _was recorded by UV-Vis absorption during a centrifugation-washing process (Figure 3). At the beginning, every vial consists of 0.5 mL of Li-MoS_2_, diluted with 1.5 mL of distilled water and sonicated for 5 min. The UV-Vis spectrum of the initial solution is shown in Figure 3a. Each vial was centrifuged for 20 min at 10,000 rpm, and the liquid part was replaced with distilled water in order to remove the excess lithium. The process has to be repeated at least five times, during which the pH is lowered from 12 to 7, and thus, the single layers are obtained from the removed liquid. The corresponding spectrum is shown in Figure 3b.

Both the Li-MoS_2 _and the exfoliated MoS_2 _have peaks around 200 nm as well as two peaks around 300 and 400 nm (4.11 and 3.11 eV, respectively), as seen in Figure 3. There is no evidence of excitons at approximately 700 nm. We suggest that the two peaks associated with the exfoliated material are A and B excitons that exhibit a large blueshift (2.23 and 1.05 eV, respectively) due to the quantum-size confinement. To assert this claim, the effective mass treatment was applied [22]. This model is used to describe size quantization of the carriers' energy spectrum in semiconductors. In terms of the model, and in the size regime where quantum confinement effects are prominent, the shift of the absorption edge or bandgap is inversely proportional to the effective mass of the excitons:

$$\text{Δ}E_{g}\approx \frac{{\left(\pi \hbar \right)}^{2}}{\mu L_{\text{z}}^{2}},$$

where Δ*E*_g _is the energy shift, *μ *is the excitons' effective mass in the direction parallel to the *z*-axis, and *L*_z _is the thickness of the nanoparticles in the *z*-direction.

For the given exciton masses, *μ*^A ^= 1.28 *m*_e _and *μ*^B ^= 4.10 *m*_e _[23], and energy shift obtained in our experiment, we can estimate the thickness of the sample: $L_{\text{z}}^{\text{A}}\approx \text{ 5 Å}$ and $L_{\text{z}}^{\text{B}}\approx \text{ 4 Å}$. Both values are in the frame of accuracy for the MoS_2 _monolayer thickness [11].

The aging process of the exfoliated material was observed, as shown in Figure 3c. The main feature is that the peak at 200 nm becomes more prominent in time, but the shoulders at 300 and 400 nm remain unaltered. For the exfoliated bulk material, the evidence of restacking starts to occur in a few days with the reappearing of A and B excitons at 700 nm [11]. On the contrary, for the exfoliated nanotubes, this effect was not observed even after 3 months.

**Figure 4:**
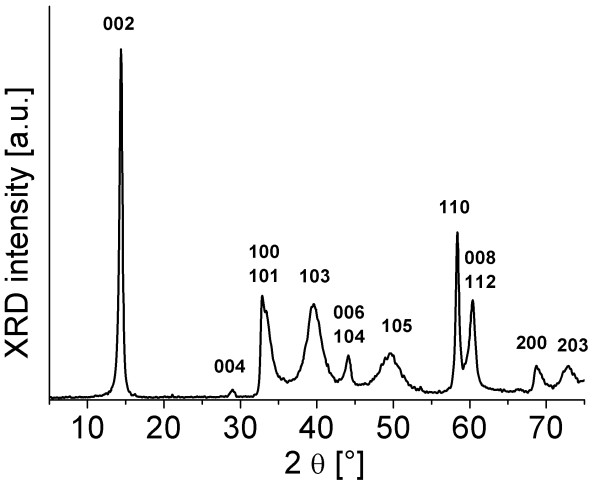
**XRD of the nanotubes**. X-ray spectrum of the MoS_2 _coaxial nanotubes.

**Figure 5:**
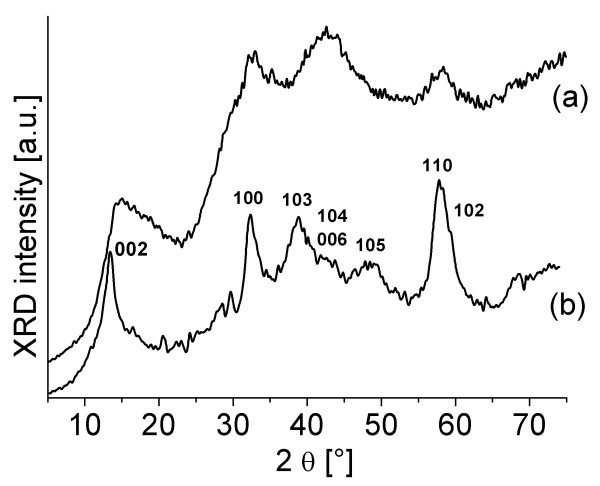
**XRD of the exfoliated nanotubes**. X-ray spectra of (**a**) the exfoliated MoS_2 _in a wet, paste-like form and (b) the material after drying.

The XRD patterns of the MoS_2 _nanotubes, exfoliated nanotubes in a wet, paste-like form, and dry, restacked MoS_2 _nanolayers are presented in Figures 4 and 5. Figure 4 shows the XRD spectrum of MoS_2 _nanotubes used for exfoliation, with the peaks indexed in accordance with the hexagonal lattice parameters after Joint Committee on Powder Diffraction Standards card number 77-1716. The spectrum of the exfoliated material was recorded in a wet, paste-like form in order to avoid drying and restacking. The absence of the (00l) peaks suggests the majority of single layers in the sample. The (002) peak is highly asymmetric with a sawtooth shape. This effect was explained as the indication of the presence of the superlattice [8]. When the sample is dried, all of the peaks characteristic for 2H-MoS_2 _reappear.

To quantify the effective size of the particles for the nanotubes and wet, paste-like MoS_2_, the broadening of the XRD lines is considered by applying the Debye-Scherrer equation:

$$L=\frac{K\lambda }{\beta Cos\theta },$$

where *L *is the effective particle size, *K *is the shape factor, *β *is the XRD line broadening at half the maximum intensity given in radians, *λ *is the wavelength of X-rays, and *θ *is the scattering angle. In order to use the equation, peaks must be broadened due to crystallite size, not due to instrument optics, so the peaks that are not well resolved are not taken into account. The results are summarized in Table 1.

**Table 1:** The calculated effective particle size of the given reflections

Reflection	*β* (rad)		Effective particle size (nm)	
	MoS_2 _nanotubes	Exfoliated MoS_2_	MoS_2 _nanotubes	Exfoliated MoS_2_
(002)	0.01	0.08	15 ± 1	2 ± 1
(100)	-	0.02	-	7 ± 1
(103)	0.04	0.07	5 ± 1	3 ± 1
(105)	0.05	0.08	5 ± 1	3 ± 1
(110)	0.01	-	30 ± 1	-
(200)	0.03	-	15 ± 1	-

## Conclusion

Exfoliated MoS_2 _coaxial nanotubes are produced via chemical exfoliation, resulting in single-layer flakes that are stable for months, with a low degree of restacking. Both X-ray spectra and TEM images confirm that the material is indeed composed of MoS_2 _monolayers. In addition, UV-Vis spectra show a strong quantum confinement effects. The relatively simple process of getting one-layer-thick MoS_2 _can be used to provide new types of materials with possible applications in polymer composites, photovoltaics, and nanoelectronics.

## Competing interests

The authors declare that they have no competing interests.

## Authors' contributions

BV acquired and interpreted the data, carried out the analysis and Uv-Vis measurements, and drafted the manuscript. RD helped in the chemical part by carrying out the lithium intercalation. MKG and NH participated in the additional UV-Vis measurements. SDS acquired the XRD data. MR has been involved in revising the manuscript and has given the final approval of the version to be published. All authors read and approved the final manuscript.
